# Microstructural and Electrochemical Study: Pitting Corrosion Mechanism on A390 Al–Si Alloy and Ce–Mo Treatment as a Better Corrosion Protection †

**DOI:** 10.3390/ma17123044

**Published:** 2024-06-20

**Authors:** Héctor Herrera Hernández, Araceli Mandujano Ruiz, Carlos Omar González Morán, José Guadalupe Miranda Hernández, José de Jesús Agustín Flores Cuautle, Jorge Morales Hernández, Irma Hernández Casco

**Affiliations:** 1 Laboratorio de Investigación en Electroquímica y Corrosión de Materiales Industriales, Universidad Autonoma del Estado de Mexico, CU Valle de Mexico, Blvd. Universitario s/n, Predio San Javier, Atizapan de Zaragoza 54500, Mexico; hherrerah@uaemex.mx (H.H.H.);; 2 Laboratorio de Investigación de Materiales y Procesos Inteligentes, Universidad Autonoma del Estado de Mexico, CU Valle de Mexico, Blvd. Universitario s/n, Predio San Javier, Atizapan de Zaragoza 54500, Mexico; 3 Laboratorio de Investigacion y Desarrollo de Materiales Industriales, Universidad Autonoma del Estado de Mexico, Blvd. Universitario s/n, Predio San Javier, Atizapan de Zaragoza 54500, Mexico; jgmirandah@uaemex.mx; 4 CONAHCYT-Tecnologico Nacional de Mexico, Instituto Tecnologico de Orizaba, Oriente 9, Orizaba 94320, Mexico; 5 Centro de Investigación y Desarrollo Tecnológico en Electroquímica, Parque Tecnologico Queretaro s/n, Sanfandila, Pedro Escobedo, Queretaro 76703, Mexico; 6 UAP Tianguistengo, Ingenieria en Producción Industrial, Universidad Autonoma del Estado de Mexico, Paraje el Tejocote, San Pedro Tlaltizapan, Santiago Tianguistengo 52640, Mexico

**Keywords:** Al–Si alloys, sulfuric anodizing treatment, Ce–Mo surface modification, electrochemical impedance measurements, oxide films, pitting corrosion

## Abstract

Sulfuric acid anodizing assisted by a hydrothermal sealing with inhibitors [Ce^3+^-Mo^6+^] was used to prevent pitting corrosion on spray-deposited hypereutectic Al–Si alloy (A390). An investigation concerning the evaluation of pitting corrosion resistance on the anodic oxide thin film with ions incorporated was carried out in NaCl solution using electrochemical measurements (i.e., potentiodynamic polarization and electrochemical impedance spectroscopy, EIS). The influence of Si phase morphology and size on the growth mechanism of an anodic oxide film was characterized by scanning electron microscopy (SEM) and X-ray diffraction (XRD). The results were then compared with those for its equivalent IM390 alloy (Al-17Si-4.5Cu-0.6Mg) produced through a conventional process ingot metallurgy, IM. The electrochemical findings indicate that sulfuric acid anodizing followed by a simple hot water sealing treatment was ineffective. In this manner, an intense attack was localized by pitting corrosion that occurred on the anodic oxide film in less than three days, as denoted by characteristic changes in the EIS spectra at the lowest frequencies. Improved results were achieved for Ce–Mo surface modification, which can provide better corrosion resistance on the aluminum alloys because no signs of pits were observed during the corrosion testing.

## 1. Introduction

A390 casting alloy is a typical aluminum–silicon alloy used for many years in automotive engine components, particularly in cylinder blocks, pistons, or connecting rods. In recent years, it has been used in pumps, compressors, and transmission components, and in various applications requiring low density, excellent wear and corrosion resistance, wear resistance, strength at high temperatures with low thermal expansion, and machinability [1,2,3]. Due to its attractive properties, persistent attempts have been made to replace gray cast iron and many other conventional ferrous materials by lightweight aluminum alloys, such as A390, which is still considered a promising material for automotive engine parts. In this alloy, silicon is the primary alloying element and is responsible for conceding mechanical strength to the soft Al matrix due to the precipitation of harder silicon particles dispersed throughout the entire eutectic Al matrix, providing a similar appearance to that of reinforced metal matrix composites (MMCs). However, some additions of copper (Cu) and magnesium (Mg) make the alloy heat-treatable for additional hardening through Al_2_Cu and Mg_2_Si precipitation as second-phase compounds [4,5]. However, these elements go into a solid solution during alloy manufacturing, commonly precipitating within a large, fragile, fibrous, and coarser grain structure under slow solidification rates. This condition is a typical overview of the resulting microstructure of the ingot metallurgy (IM) process [6,7].

Furthermore, IM is the current technology in manufacturing high-Si-content aluminum alloys for engine components at a reasonably low cost. In addition, IM is associated with the formation of large and coarser primary Si phase particles ranging from 50–150 µm when Si solidifies in the Al matrix at lower cooling rates. Subsequently, it mixes with inter-dendritic eutectic phases and hard insoluble intermetallic compounds. These microstructural features could degrade the mechanical properties of these materials and also increase the susceptibility to pitting corrosion damage, and limit their application in diverse potential fields [8,9,10,11].

Over the last two decades, Estrada and Duszczyk [6,12], Gupta and Lavernia [7,13], and, more recently, Chen et al. [14] and Chiang and Tsao [15] have demonstrated that increasing the solidification rate over 10^2^ to 10^3^ °C/s brings a significant microstructural modification in distribution, morphology, and size of the Si particles in the matrix, often compared to those found in conventionally processed materials. Therefore, various technologies that employ rapid solidification processes (RSPs), such as splat quenching [16,17], atomization [18,19], and melt spinning [20,21], have become very influential in attaining microstructural refinement. Atomization with water is the most useful technology for low-cost aluminum powder production. However, its application is limited for alloys that tend to react violently with water. This reaction leads to the growth of an amorphous oxide layer on the powder surface with adsorbed hydrogen gas, which results in high detriment to the mechanical properties during powder consolidation and hot working [22]. Internal porosity, surface cracking, delamination, or blistering may also occur due to the hydrogen evolution reaction when the material is exposed to heat treatment at 470 °C for 1.5 h or during high-temperature service [6,19]. Given these limitations, neither powder metallurgy (PM) nor conventional ingot molding (IM) methods are good options for processing reactive materials.

Alternatively, spray atomization coupled with deposition technology (SD process) seems to be a suitable route for manufacturing hypereutectic Al–Si alloys. This technology is a relatively new metallurgical process that involves the energetic disintegration of a molten metal stream with a high-velocity gas jet, producing a stream of micro-sized droplets in an enclosed protective atmosphere chamber. With this in mind, an enclosed chamber under a nonreactive atmosphere is necessary to avoid surface oxidation of the atomized droplets. The molten spray droplets generated lose their heat during flight or sudden extraction after contact with a rotative cold substrate, which leads to a controllable build-up into a high-density deposit with a required shape and dimensions named preform or deposit [12,20,23,24,25,26,27,28]. Spray atomization and deposition technologies (SD-PROCESS) have received considerable attention as a viable alternative route to produce materials with heterogeneous particle distribution based on the concept of rapid solidification processes (RSPs), such as synthesizing the Mg and Al; and obtaining discontinuously reinforced metal matrix composites (MMCs) [7,23,29,30,31,32,33,34,35,36,37]. Powder metallurgy, spray-atomization, and deposition can produce materials with similar attractive combinations properties; however, these have, along with a reduced number of processing steps, the possibility of near-net-shape manufacture without the disadvantages of secondary treatments (degassing or consolidation).

It has been reported [38,39,40,41,42,43,44,45] that aluminum resists some corrosive attacks due to its inherent oxide film that allows it to grow homogeneously onto the surface during exposure to aggressive atmospheres. However, this film does not offer sufficient protection for Al–Si alloys, because of the presence of Si particles that disrupt the oxide film’s continuity. Therefore, a strong microgalvanic coupling exists over the entire exposed surface that leads to highly localized attack, particularly in applications involving wet and salty environments [41,44]. The sulfuric acid anodizing process is frequently used to promote further protection of aluminum alloys [45]. For a long time, treatments like anodizing based on chromates [46] were used as corrosion protection for high-strength Al alloys used in severe corrosive conditions. Therefore, the anodizing treatment has been widely used as an electrochemical process to convert the aluminum surface into a thicker aluminum-rich oxide film compared to that formed naturally for effective corrosion control. A chromic acid bath (H_2_CrO_4_) or potassium dichromate salts (K_2_Cr_2_O_7_) containers are frequently used for industrial protective treatments.

Recently, several environmental and health legislations have limited the use of chromium compounds due to their high toxicity and carcinogenic nature [42,47,48,49,50], which leads to the urgent search for new and better alternative methods [51,52,53,54,55,56,57,58,59]. For this reason, sulfuric acid anodizing treatment followed by a cerium–molybdenum surface modification process was investigated through this research, to increase the corrosion protection and durability in anodized A390 Al–Si alloy produced under spray-deposited technology.

Mansfeld and coworkers [43,60,61,62,63,64,65,66] investigated the corrosion protection of aluminum alloys by the cerium–molybdenum surface modification process; they reported significant improvement in the resistance to localized corrosion. Mansfeld et al. [64,67,68,69] also examined the effects of chemical passivation on the corrosion behavior of aluminum alloys by immersion in a cerium chloride (CeCl_3_) solution. Xingwen and Chunan [70] used cerium salts as a sealing treatment for anodized Al2024 alloy. However, several researchers reported that sulfuric acid anodizing is applied to commercial aluminum alloys; most of these publications are focused on the microstructure and physical properties [71,72,73,74,75]. Thus, only a few reports are known about to the electrochemical impedance spectroscopy (EIS) behavior, applied to spray-deposited hypereutectic Al–Si alloys with sulfuric acid anodizing combined with the cerium–molybdenum modification process. This subject was the primary purpose of the present research.

## 2. Experimental Procedure

### 2.1. Materials

**Figure 1:**
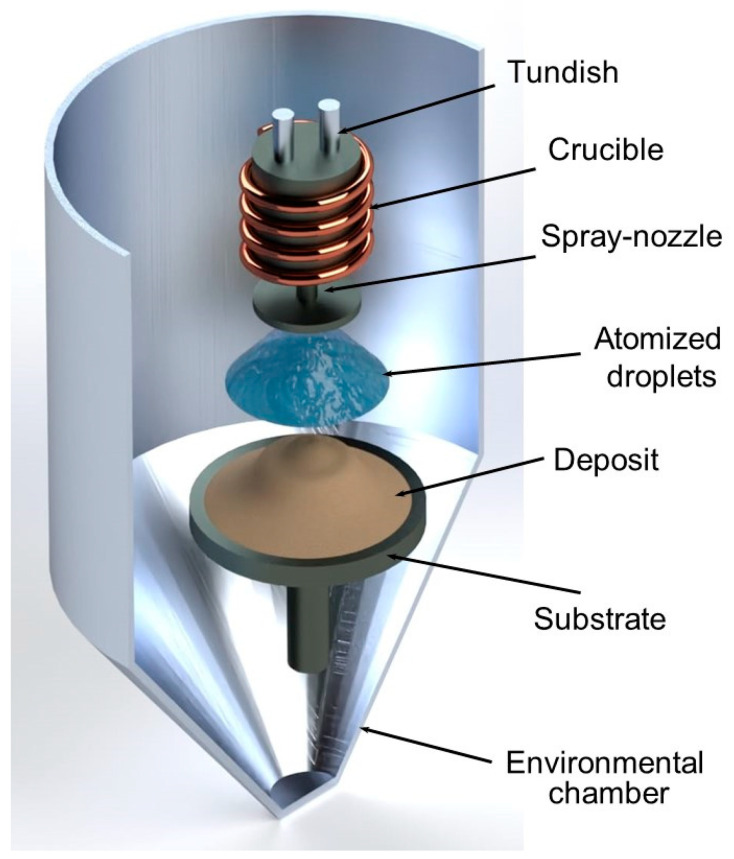
Schematic illustration of the experimental process used to produce Al–Si alloys through spray-atomization and deposition technology at the University of California, Irvine UCI [75,76].

Spray-atomization and deposition technology was used for the synthesis of Al–Si materials [25,26,76,77] with equipment installed at the University of Irvine California (UCI), (Department of Mechanical Engineering), with the following description: (i) The alloy was heated to the pouring temperature of 800 °C at a graphite crucible in a protective environment at 1 atmosphere of pressure; (ii) the molten alloy was placed into an atomizer through a ceramic delivery tube, and then it was finely dispersed into micrometer-sized droplets using a nitrogen gas jet at a pressure of 3.1 MPa. The gas–metal ratio (GMR) was about 2.5 or 4 m^3^/kg, which resulted in different thermal conditions of the sprayed materials. Finally, (iii) the partially solidified droplets were collected on a water-cooled rotating metallic (Cu) substrate (45 rpm) that was hydraulically controlled and placed 46 cm away from the atomizer nozzle. A coherent deposit was collected from the molten droplets named preform or deposit. Figure 1 depicts a schematic representation of the equipment used for the spray-atomization and deposition process.

**Figure 2:**
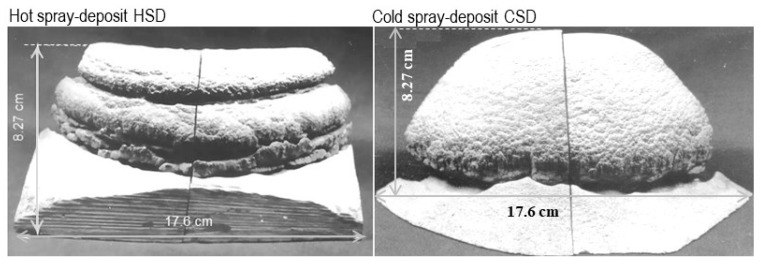
Transversal section of spray deposits of hypereutectic Al–Si alloy produced under different thermal conditions.

Two sprayed deposits with a bell shape were produced at UCI: one at 2.5 m^3^/kg gas-metal ratio, named “HSD” (hot-spray deposit), and the other at 4 m^3^/kg, named “CSD” (cold-spray deposit); both deposits have identical dimensions of 17.6 cm in diameter and 8.27 cm in length, as shown in Figure 2. These preforms were mechanically sectioned from their central area to cut cylindrical billets of 2.54 cm in diameter and 8 cm in length, to ensure a completely fine and uniform structure with a lower porosity content. The billets were hot-extruded at 480 °C with a 4:1 reduction ratio at 5 mm/s ram speed; after that, these were air-cooled for 12 h. As a result, two extrudate products were obtained: ExHSD was extruded directly from the HSD billet, whereas ExCSD was obtained from the CSD billet. Table 1 summarizes the information on the Al–Si spray deposits and their extrudates. Representative samples for microstructural characterization, electrochemical study, and anodizing treatments were removed by sectioning the preforms and extrudates.

### 2.2. Microstructural Characterization

Table 2 shows the chemical composition given in wt% of the spray-deposited aluminum alloy and its extrudates, which were obtained by X-ray fluorescence (XRF) (XRF gun analyzer, S1 Titan with 50 KeV X-ray tube, Bruker, Mannheim, Germany). These materials generally contain approximately 17% silicon as a significant addition, which provides hard mechanical strength with wear resistance through hardening precipitation. They also contain copper (~4.5% Cu), magnesium (~0.55% Mg), and iron (<0.5% Fe) to make the alloy heat treatable; this composition is very close to that of the casting IM390 alloy (16.7 wt% Si, 3.4 wt% Cu, 0.56 wt% Mg, 0.7 wt% Fe), which is given in bars of 2.54 cm in diameter and was used as the reference material in this research work. The phases present in the as-sprayed deposits and their extrudates were identified by XRD, accomplished in a Siemens D5000 diffractometer using Cu-Kα_1_ radiation λ = 1.54056 Å. The XRD patterns were obtained between 20° and 90° in the 2θ diffraction angle with a resolution of 0.02° and a time step of 1 s. The morphological and microstructural characteristics were studied by scanning electron microscopy (JEOL SEM-electron microanalyzer, Tokyo, Japan) using a JEOL JXA-8200 equipped with an electron probe microanalyzer (EPMA). The samples were prepared and polished using standard metallographic procedures and etched using a Keller’s reagent (2.5% HNO_3_: 1.5% HCl: 1% HF: 95% H_2_O) to reveal the Si precipitation. The quantitative image analysis software was used to measure the Si particle size distribution.

### 2.3. Anodizing Process

Before anodizing, the samples were wet-abraded with an abrasive paper #600 grit and then chemically degreased in an alkaline bath (50 g/L NaOH) for 10 min at 45 °C. The following process was etching in a concentrated acid solution (H_2_SO_4_) at 70 °C for 20 min to remove corrosion products. After surface cleaning, the samples were washed in distilled water and then air-dried.

Moreover, anodizing treatment was performed with a current density of 27 mA/cm^2^ in 180 g/L H_2_SO_4_ (95%) at room temperature for 30 min. Subsequently, the samples were cleaned using distilled water and treated immediately by one of the following sealing procedures:

(i) Hot water sealing (HWS): Sealing with distilled water was conducted at near-boiling temperature for 60 min.
(ii) Cerium surface modification process (CeSM): The samples were dipped in boiling CeCl_3_ solution (10 mM) for 20 min.
(iii) Cerium–molybdenum surface modification process (Ce–MoSM): Two solutions were used to submerge the samples: boiling in CeCl_3_ solution (10 mM) for 20 min then rinsing and submerging in boiling Na_2_MoO_4_ solution (0.1 M) for 20 min.

After treatments, the anodized samples were dried immediately under an air stream at room temperature and stored in a desiccator container to keep them dry for the next electrochemical testing.

### 2.4. Electrochemical Test Methods

The electrochemical behavior of Al–Si alloys was evaluated in 3.5% NaCl solution at room temperature, both in the as-received condition and after surface treatment by anodic polarization and electrochemical impedance spectroscopy (EIS) (IM6-Zahner, Kronach, Germany) measurements. EIS has an essential advantage over other electrochemical techniques because it is a nondestructive tool that becomes suitable for studying coatings for metal corrosion protection over extended periods [78]. The samples were encapsulated by epoxy resin with a copper wire connector, and with an exposed area of 1 cm^2^ as a working electrode (WE). After curing, the exposed surface was grinded with SiC emery paper (numbers 80, 120, 220, 340, 400, and 600 grit) and polished with alumina particles of 0.5 µm diameter. All experiments were conducted at the open-circuit potential (OCP) in a conventional three-electrode cell employing a saturated Ag/AgCl electrode as reference electrode (RE) and a cylindrical SS316L as a counter electrode (CE). Before measurements, the test electrode was kept in the solution at the corrosion potential (E_corr_) for almost 15 min to reach an equilibrated state. Afterward, anodic polarization curves were recorded using a PARSTAT-4000 (S/N 14181860-DR3H) from −1400 to +400 mV (Ag/AgCl) using a scan rate of 1 mV/s. The experimental EIS data were acquired at the OCP using a workstation IM6-Zahner (S/N IM6-12450-DR3H) frequency response analyzer (FRA) (IM6-Zahner, Kronach, Germany) which was controlled by a personal computer using the Thales XT-v.21 software package. The frequency range examined was from 100 KHz to 1 mHz with a voltage perturbation amplitude of 10 mV at 10 points per decade acquisition rate. The experimental impedance data were used to obtain information concerning surface properties, where the impedance information was fitted to an appropriate equivalent electrical circuit (EEC) model using the software package ANALEIS developed by Mansfeld et al. [43,44,79]. Furthermore, fluctuation transients in potential (mV) were also recorded at OCP condition for 60 min at each immersion time tested. EIS tests were periodically used at increasing periods of time to monitor the evolution of electrochemical behavior. Finally, the surface appearance after corrosion tests was investigated through SEM examination.

## 3. Results and Discussion

### 3.1. Microstructure Evaluation of Spray-Deposited Al–Si Alloys

**Figure 3:**
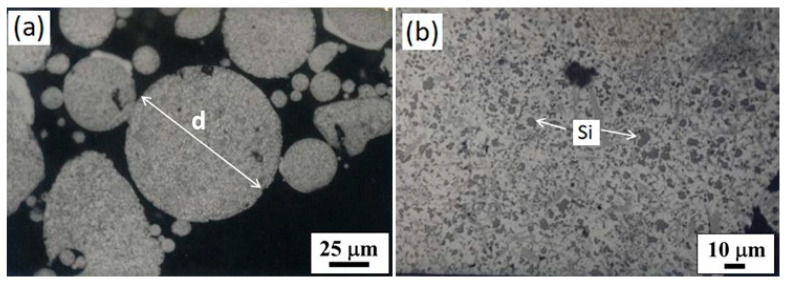
Optical micrographs of the cross-section of Al–Si powder produced by nitrogen gas atomization show a spherical shape of the powder particles (**a**) and fine silicon precipitation in the aluminum matrix (**b**).

Figure 3 shows the typical morphology of a single particle and the internal solidification microstructure of the atomized Al–Si powder with nitrogen gas. According to Figure 3a, most of the powder particles exhibit a spherical shape with a small cluster of particles attached. This morphology results from continuous collisions among liquid or semiliquid coarse particles with fine solidified particles in a turbulent atmosphere. It is well known that the final shape of the metal particles is strongly dependent on the solidification behavior of the droplets and the powder production technique [3,6,13,18,19,20,21]. During atomization with gas, the molten metal stream breaks into liquid waves due to momentum transfer from the gas to the liquid. Molten metal is also fragmented by the shock of atomizing gas into ligaments that eventually acquire a spherical shape under the action of high surface tension forces.

On the other hand, the progress of the microstructural details during the solidification of the nitrogen-atomized Al–Si powder was observed by optical microscopy with samples prepared by a standard metallographic procedure, as shown in Figure 3b. This micrograph shows a cross-section of a group of Al–Si particles mounted on an epoxy resin, and then polished, after being superficially etched with a 1.5% nitric acid (HNO_3_) solution known as Keller’s reagent. The microstructure of each powder particle consists of a uniform dispersion of fine primary Si particles (shown in dark gray color) in the α-Al matrix (shown in white color); these particles appear to be block-like or spheroidal in shape, which is common morphology in rapidly solidified powders. Therefore, based on the rapid solidification process concept [6,12,35], eutectic phases, coarser Si particles, needle or flake-like shape of Si phase, and hard precipitation of intermetallic compounds were completely suppressed in the structure of rapidly solidified Al–Si powders.

Consequently, the deposition of the molten metal droplets onto the surface of the metal substrate occurs when the surface temperature is low and at a suitable distance to guarantee that all sprayed droplets are totally solidified before the deposition process. Moreover, these conditions also lead to each separate droplet retaining its own identity and microstructural characteristics after deposition. However, some droplets may reach the surface in an almost entirely liquid state and impact on a semiliquid surface, which causes the extension of the liquid flow of the molten metal on the preform surface. Initially, these droplets are impacted at high velocity onto the preform, resulting in dendrite fragmentation.

**Figure 4:**
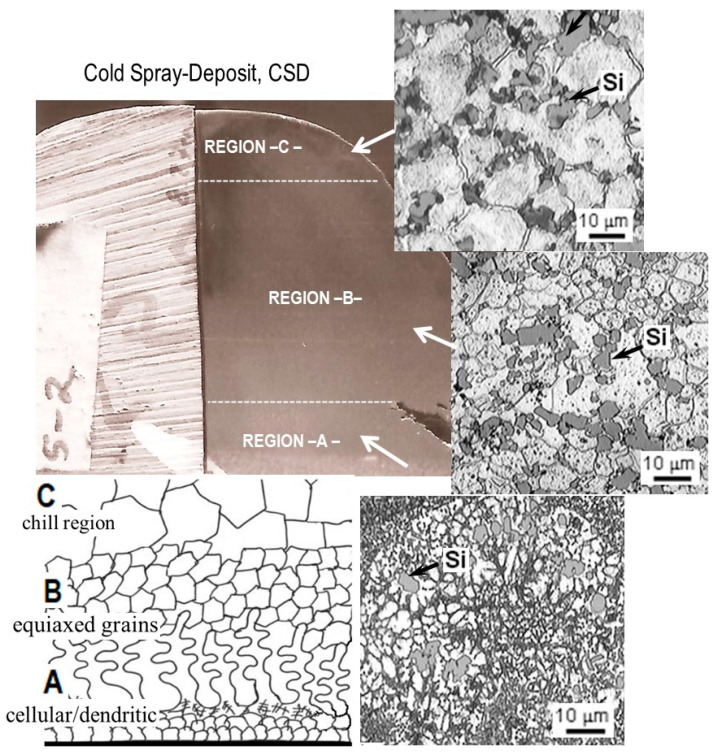
Cross-sectional view of a cold-spray-deposited (CSD) Al–Si alloy showing the microstructure of three regions: Region “A” is adjacent to the substrate surface. Region “B” is the central portion. Region “C” is near the upper surface.

Thus, these pre-solidified and deformed particles lose their identity in that semisolid/semiliquid layer and, in combination with coarse fully molten droplets, solidify to form a solid homogeneous preform with small equiaxed grains plus a mixture of dendritic fragments. Therefore, as a result of variations in cooling rates and thermal conditions during the deposition stage, the preform structure can solidify in different microstructural regions with changes in the surface quality, as suggested by X. Liang and E.J. Lavernia [26,27]. They observed in their research work significant variations in microstructural features whereby the microstructure critically depends on location in the spray-deposited material (preform) due to the heat transfer mechanism. According to this experience, the CSD preform sprayed at the cold condition (4 m^3^/kg of gas–metal ratio) was sectioned into three diverse regions: “A-bottom”; “B-middle”; and “C-top”, as is shown in Figure 4.

The region “A” (the bottom of the preform) is positioned near the collecting Cu substrate. According to the microstructural results, the region “A” is composed of a cellular/dendritic structure, as is clearly shown in the micrograph of Figure 4 (region “A”). The dendrites grow up at the substrate interface in a perpendicular direction to the substrate (i.e., opposing the heat transfer direction). The region away from the collecting substrate is region “B”, which refers to the central portion of the preform; its microstructure is uniform, which is composed of a fine dispersion of Si particles and equiaxed grains of α-Al phase. Small microporosity is also observed; based on these characteristics, this region is relatively dense, near the theoretical density (97–99%).

Finally, the region “C” comprises the preform’s upper surface, also known as the chill zone. The microstructure in this region is coarser with macropores, where equiaxed grains are not well defined. In particular, these results indicate that small droplets are easily carried and accelerated by the component of the radial velocity of the gas-atomized stream along the center line of the atomized spray, leading the droplets to solidify rapidly in a fine equiaxed grain structure with well-defined grain boundaries, such as that observed in region “B”. For research purposes, in this research work, some representative cubic samples of 1 cm × 1 cm × 0.5 cm were taken from the central portion (region “B”) of the spray-deposited (either preform CSD or HSD) material in which the microstructural features are more uniform than those on regions “A” or “C”.

**Figure 5:**
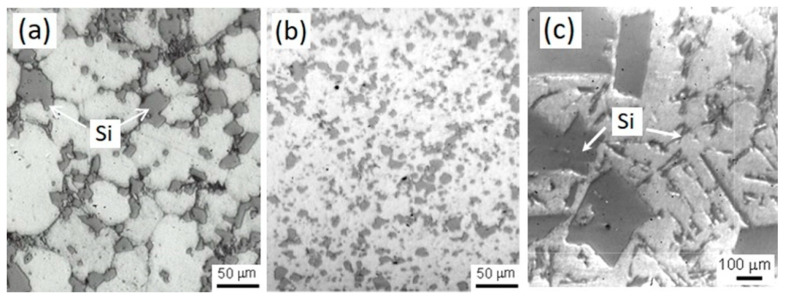
Micrographs of Al–Si preforms showing (**a**) grains morphology and Si particles of as-sprayed deposit HSD, (**b**) fine uniform precipitation as-sprayed deposit CSD, and (**c**) Si and eutectic phase in IM390 ingot alloy.

As can be seen in Figure 5, the silicon particles depend strongly on the ratio between the mass flow rate of gas and the mass flow rate of melt (gas/metal ratio, GMR), which dominates the thermal condition in spray deposition (high GMR is referred to deposits sprayed at the cold condition and low GMR for hot-spray condition). The distribution, size, and morphology of Si particles in the spray-deposited Al–Si alloy, as a function of GMR, are shown in Figure 5a,b, respectively. A coarser microstructure with primary block-like Si particles contacting each other (agglomeration in microzones) is clearly observed in the preform sprayed under hot conditions (HSD, 2.5 m^3^/Kg) with average size of silicon particles of about 50 μm.

Therefore, the convection effect for hot-sprayed deposits may cause the coarsening of Si particles. However, the preform CSD (cold-spray condition, 4 m^3^/Kg) shows a finer distribution of Si particles with a size of less than 10 μm. In addition, small micropores were also observed in the deposited Al–Si materials. Therefore, the cooling and solidification conditions are the driving force that makes the difference in the size of primary Si particles during the deposition of the molten metal droplets, in contrast to the materials manufactured with a slow cooling rate (~10 °C/s, conventional casting), where the silicon particles are much larger and coarser (in the range of about 150 to 500 μm), as is observed in Figure 5c.

The microstructure of the IM390 Al–Si alloy frequently appears to consist of a coarser Al–Si eutectic phase and primary Si particles in needle-like form embedded in the dendritic α-Al matrix, which promotes brittleness for the alloy. This morphology of coarser Si particles, eutectic Al–Si phases, and the original dendritic structure is suppressed in the spray-deposited Al–Si materials because of a rapid solidification process; silicon morphology becomes completely block-like dispersions or spheroidal with a fine distribution in the aluminum matrix. On the other hand, the extrusion process is also an essential parameter in which the Si particles could be broken by a plastic deformation mechanism; thus, the resulting new fine particles can improve the mechanical properties such as toughness, strength, and wear resistance, and intervene by modifying the electrochemical behavior of the alloy.

**Figure 6:**
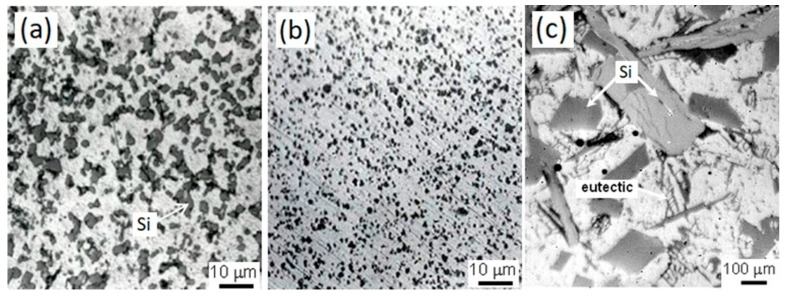
Micrographs of Al–Si extrudates showing grain morphology and Si particles; (**a**) coarser dispersion in extrudate as-sprayed deposit HSD, (**b**) fine uniform precipitation of extrudate from as-sprayed deposit CSD, and (**c**) Si and eutectic phase in IM390 casting alloy.

**Figure 7:**
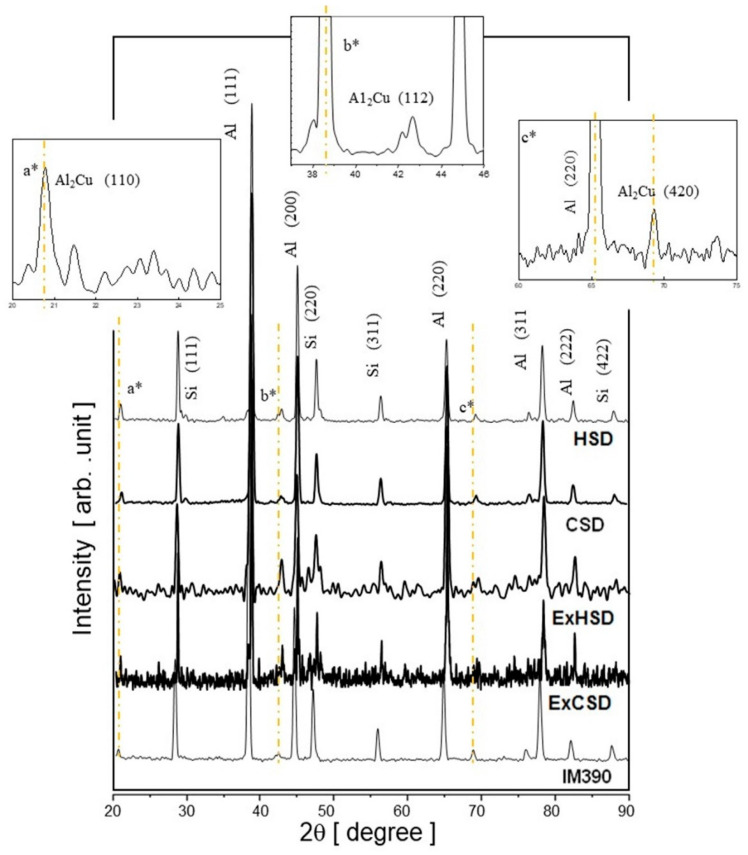
X-ray diffraction patterns of Al–Si alloy as-sprayed deposits and their corresponding extrudates products; also included is the IM390 casting alloy as reference. Insets; a* Al_2_Cu precipitates on (110) plane at 20.7°, b* Al_2_Cu precipitation on (112) plane at 38.3°, c* precipitates on (420) plane at 69.7°.

The microstructure of the extruded products shown in Figure 6 indicates closed microporosity present in the sprayed deposits HSD or CSD during hot extrusion, observing that the average size of the Si particles decreased. The average crystal size of Si for the extrudates ExHSD is ~12 μm (Figure 6a), and for ExCSD (Figure 6b), it is less than 5 μm. Therefore, the particles obtained in the sprayed deposits and their extrudates are much finer than those obtained for the ingot molding counterpart because the solidification rate of this commercial alloy is about 10 °C/s. In contrast, the solidification rate in spray deposition is between 10^3^ and 10^5^ °C/s. Materials cooled at high solidification rates increase the nucleation of Si and its solubility and also exhibit a reduction in the free energy for the growth of Si particles. Furthermore, impact deformation and fracture experienced by the solidified droplets during spray-atomization and deposition may also break up the primary Si particles formed before the deposition stage. The broken Si particles increased the number of nucleation sites at the top layer of the spray-deposited material [25,26,27,28]. The present phases in the Al–Si materials were identified by XRD and were compared with the standard diffraction files from each specific phase. Figure 7 shows typical XRD patterns for the spray-deposited materials and their respective extrudates products. Also, the same figure shows the X-ray pattern for the conventional ingot material (IM390). Two phases were identified in the α-Al matrix: Si and Al_2_Cu. Images taken by optical microscope did not make it possible to identify the Al_2_Cu phase due to the poor contrast resolution, requiring color metallographic techniques.

The XRD analysis did not indicate any possible reaction between Al and Si particles, particularly in the formation of intermetallic Al_2_Cu precipitating by the reaction of Al with traces of Cu in the alloy. It is also known that Al_2_Cu tends to react and dissolve in molten Al, leading to the formation of Al_2_Cu precipitates during the solidification. L. Lasa and J.M. Rodriguez-Ibabe [1,2,3,4] and L. Del Castillo and E.J. Lavernia [80] reported that Al_2_Cu precipitates at the grain boundaries as fine blocky particles with aspect ratios that favor dissolution in the aluminum matrix.

### 3.2. Electrochemical Measurements

#### 3.2.1. Potentiodynamic Polarization Tests of Untreated Condition

**Figure 8:**
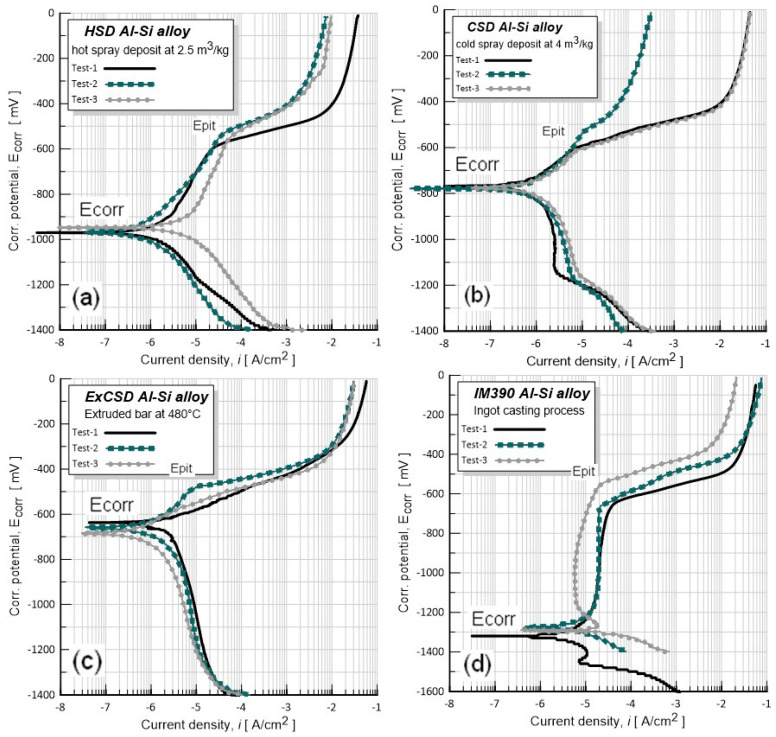
Potentiodynamic polarization curves of Al–Si alloys in 3.5% NaCl. As-received condition for the spray-deposited CSD or HSD and its extrusion product; IM390 casting alloy response is also shown as comparative behavior. (**a**) HSD hot spray deposit (2.5 m^3^/kg), (**b**) CSD cold spray deposit (4 m^3^/kg), (**c**) Extruded bar at 480° C for CSD, (**d**) IM390 ingot casting process.

Figure 8 describes the electrochemical behavior by using potentiodynamic polarization curves of spray-deposit Al–Si alloys and their respective extrudate product. The measurements were obtained at laboratory conditions in an aerated aqueous solution of 3.5% NaCl; as a comparison, the response of casting IM390 alloy is also shown.

The materials were evaluated at least two times to confirm their behavior. Furthermore, it is worth mentioning that according to the microstructural analysis that is seen in Figure 4, the CSD preform and its extruded ExCSD have the highest structural densification with a fine porosity of less than 1%; based on this observation, the electrochemical results only focus on CSD materials. There are important differences in the corrosive behavior between spray deposits and their derivative extrudates or their counterpart casting alloy IM390 due to the silicon phase morphology and size present in the Al structure. However, all the samples revealed a certain passivity domain as a consequence of the presence of a natural oxide layer, but noticeable changes in the slope of anodic curves at the pitting potential (E_pit_) of about −670 to −579 mV are observed. These changes are usually associated with an anodic dissolution mechanism, which means the disintegration process of an Al–metal matrix through pitting corrosion.

Table 3 lists the corrosion potential (E_corr_), pitting potential (E_pit_), and corrosion current density (*i*_corr_) values for Al–Si materials. Figure 8 indicates that the difference between E_corr_ and E_pit_ is about 400 mV for the as-spray-deposited material (CSD) when a voltage polarizes these in the presence of chloride ions, Cl^−^, and 700 mV for IM390 casting. However, it is well known by many researchers [11,41,42,43] that Cl^−^ can promote pitting on metals and alloys, so Cl^−^ ions are responsible for causing a local breakdown of the passive oxide layer followed by chemical dissolution of metal matrix (e.g., pitting attack and localize dissolution), which is still not clear in the literature. According to the experimental results shown in this research of Figure 8, which are considered valuable findings in the present research work due to the expertise in the use of EIS in the corrosion field, it is possible to establish, using EIS, a reasonable mechanism that can help us to understand the pitting corrosion process on Al–Si alloys during their exposure to environments containing Cl^−^ ions.

For example, when 35 g of NaCl is dissolved in 1 L water (${\mathrm{NaCl}}_{\left(s\right)}+H_{2}O_{\left(l\right)}\;\to {\left[\mathrm{Na}\right]}^{+}+{\left[H\right]}^{+}+{\left[\mathrm{Cl}\right]}^{-}+{\left[\mathrm{OH}\right]}^{-}$), the polar water molecule has the required energy to break the ionic bond of the NaCl crystal. This process is known as dissociation, which leaves four types of ions with different charges to move them freely into the aqueous solution; two positive and two negative; the H^+^ $\left(2H+2e^{-}\to H_{2}\right)$ and Na^+^ are attracted by cathode sites, while Cl^−^ $\left(2{\mathrm{Cl}}^{-}-2e^{-}\to {\mathrm{Cl}}_{2}\right)$ and OH^−^ ions are attracted by anode sites, but only chloride ions can be charged on the interface. In this sense, metal exposed to aqueous solutions containing Cl^−^ increases the susceptibility to pitting because this kind of ion is readily combined into the atomic structure of the passive oxide film (anodic site) through a physical adsorption process via the access paths on the film (e.g., defects, cracks, or pores) [81,82]. These ions are potentially diffused into the aluminum hydroxide film (Al_2_O_3_·OH) and react with atoms of Al^+^ that compose the structural lattice of the film, displacing OH^−^ ions, hence resulting in a local failure of the oxide film and loss of passivity. In this way, Cl^−^ reaches the unprotected surface of the metal matrix and reacts with it by forming salt compounds of aluminum hydroxyl chlorides like Al(OH)Cl_2_, Al(OH)_2_Cl, or AlCl_3_ [41,42,43,44]. Concerning Graedel [83] and Brockis [84], this chlorination process occurs as follows; during the electrolyte absorption, aluminum oxide (Al_2_O_3_) is highly hydrated to boehmite (AlOOH) and subsequently transformed into bayerite [Al(OH)_3_], as described in the following reactions:

(1) $${\mathrm{Al}}_{2}O_{3}+H_{2}O_{\left(l\right)}\to \mathrm{AlOOH}\to \mathrm{Al}{\left(\mathrm{OH}\right)}_{3}$$

(2) $$\mathrm{Al}{\left(\mathrm{OH}\right)}_{3}+{\mathrm{Cl}}^{-}\to \mathrm{Al}\left(\mathrm{OH}\right){\mathrm{Cl}}_{2}+{\;\mathrm{OH}}^{-}$$

(3) $$\mathrm{Al}{\left(\mathrm{OH}\right)}_{2}\mathrm{Cl}+{\mathrm{Cl}}^{-}\to \mathrm{Al}\left(\mathrm{OH}\right){\mathrm{Cl}}_{2}↓+{\;\mathrm{OH}}^{-}$$

(4) $$\mathrm{Al}\left(\mathrm{OH}\right){\mathrm{Cl}}_{2}+{\mathrm{Cl}}^{-}\to {\mathrm{AlCl}}_{3}↓+{\;\mathrm{OH}}^{-}$$

**Figure 9:**
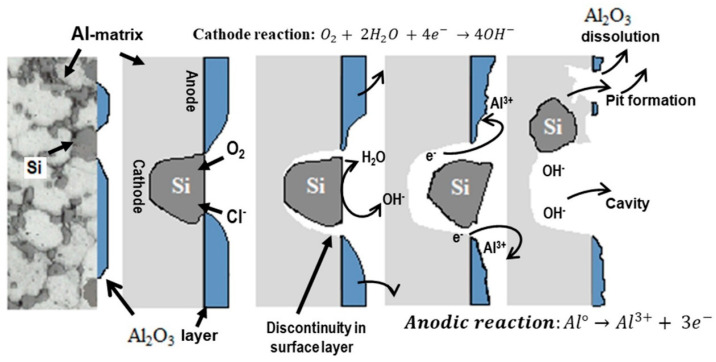
Schematic illustration of the Al/Si galvanic coupling that causes the mechanism of pitting corrosion in Al–Si alloys in 3.5% NaCl.

Once the oxide film is sufficiently thin, rapid dissolution of the oxide/metal interface occurs, and the pits are initiated spontaneously. Afterward, pitting corrosion advances due to the saturation of Al(OH)Cl_2_, Al(OH)_2_Cl, or AlCl_3_ compounds/salts inside the pits that produce a more acidic environment, which affects a possible repassivation process inside the pits [81,82,83,84,85]. For this reason, the rate of pits propagation is considered autocatalytic, so it depends on the O_2_ and Cl^−^ concentration in the aqueous solution and the pH value inside the pits. This condition suggests that all aluminum materials tested tend to form an aluminum oxide film as natural corrosion protection of the surface [80]; the process of this mechanism is graphically explained in a detailed manner in Figure 9.

Si particles on the Al matrix are identified as cathode sites and contribute to the oxygen reduction reaction ($O_{2}+2H_{2}O\;+4e^{-}\;\to 4{\mathrm{OH}}^{-}$) increasing the OH^−^ concentration on the Al surface. This reaction mainly promotes the dissolution of the defective protective oxide film, causing a prompt initiation of pits on the Al matrix. This process of aluminum dissolving through silicon particles is known as galvanic corrosion or pitting corrosion (localized corrosion). In this sense, the CSD materials were composed of fine Si particles (~12 or 5 μm) almost spherical in shape morphology and uniformly distributed on the Al matrix, in comparison with its corresponding counterpart casting alloy (IM390), which exhibits the coarsest silicon crystals as segregated in the form of needles or blocks (>150 μm).

**Figure 10:**
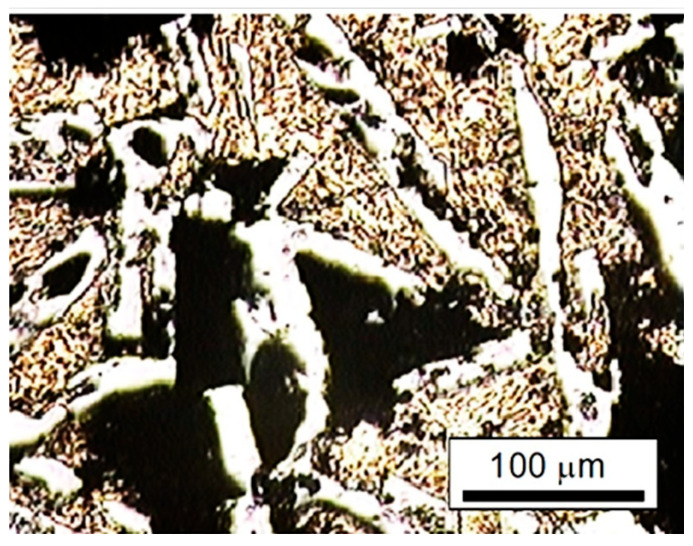
Surface appearance showing galvanic corrosion mechanism on as-casted Al–Si alloy (IM390) after exposure to 3.5% NaCl, as received condition.

Figure 8 shows that the E_corr_ of extruded ExCSD material is more positive than the spray-deposited HSD, which decreases activity from −1210 to −966 mV and increases slightly in E_pit_ from −579 to −587 mV. However, the corrosion current density (I_corr_) associated with ExCSD is almost ten times smaller (1.2 to 2.47 μA/cm^2^) than the corresponding spray-deposited HSD (14.05 μA/cm^2^) and 24 times (24.7 μA/cm^2^) its corresponding counterpart casting alloy (IM390; represented by a dotted line in Figure 8); these results indicate the best performance of spray-deposited Al–Si alloy. Nevertheless, there is a slight tendency of the gas–metal ratio GMR (4 m^3^/kg) to reduce the activity and the corrosion rate (preform, CSD) concerning that of the corresponding HSD preform (2.5 m^3^/kg). Furthermore, it is suspected that this difference in corrosion behavior is due to the dispersion of diverse silicon crystal sizes on the Al matrix; that being so, with coarser Si particles and forming eutectic phases, in addition to the nonuniform precipitation of Al_2_Cu dispersions, makes the alloy corrode more severely and easily than materials processed by spray deposit technology. An example is the illustration shown in Figure 10.

#### 3.2.2. Electrochemical Impedance Test (Untreated Condition)

##### Pitting Corrosion Behavior

**Figure 11:**
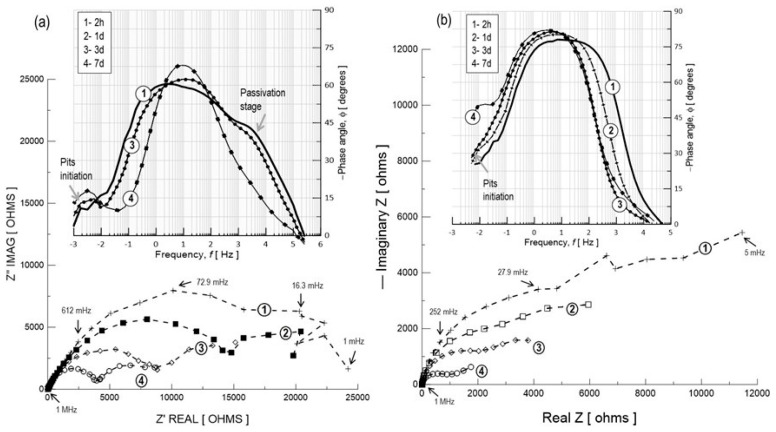
EIS spectra in Nyquist form of as-received Al–Si alloys immersed in 3.5% NaCl with respect to the exposure time. (**a**) Cold-spray deposit CSD and (**b**) IM390 alloy. Insets show a detailed view of pit initiation frequencies as Bode plots.

AC impedance spectroscopy measurements were also carried out to obtain information about the pitting corrosion process associated with galvanic coupling in aluminum–silicon alloys because its study using only the potentiodynamic polarization technique is limited [41,42,43]. Figure 11 shows the typical impedance spectra (complex plane plots: real component, Z’ vs. imaginary component, Z’’) for the untreated Al–Si materials, spray deposit CSD (Figure 11a) and its counterpart IM390 casting alloy (Figure 11b), during continuous exposure to 3.5 wt.% NaCl solution at different period of time. It is shown in Figure 11a,b that a single well-defined capacitive semicircle with a magnitude of Z’ about 2.5 × 10^4^ Ω-cm^2^ indicates that the spray deposit CSD and the ingot IM390 alloy have similar electrochemical behavior at two hours of exposure. This EIS response suggests that the charge transfer processes are impeded by the presence of an interface between the metal surface and the electrolyte, which makes it possible to assume that it represents the response of a natural aluminum oxide layer (Al_2_O_3_) that had formed during the material storage (like a passive steady stage). However, the estimated diameter of the semicircle at higher frequencies for the CSD material is larger than the cast IM390 material [86,87,88].

**Figure 12:**
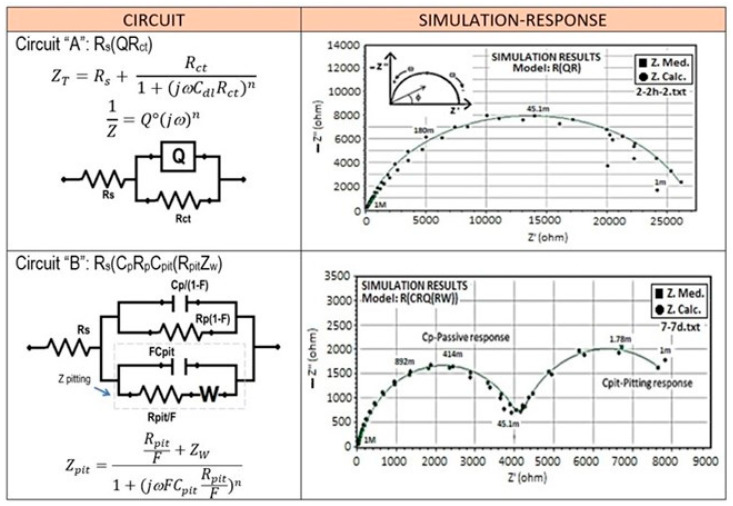
Representation of the simulation response of the equivalent electrical circuits (EECs) used to model the impedance behavior of Al–Si materials exposed for 7 days in 3.5% NaCl.

The high-frequency response suggests that the resistance of the passive surface to the charge transfer is over 10^4^ Ω-cm^2^ and could not be measured in the range of frequencies tested. This result can be explained due to the differences in the microstructural features seen before, in which the Si particle size, morphology, and also their distribution on the Al matrix represents an essential role in the continuity of the passive oxide film. The electrochemical parameters associated with this passive surface can be obtained by modeling the experimental data with an equivalent electrical circuit (EEC), proposed in Figure 12 (circuit “A”). The arrangement of circuit “A” is simple: a resistor, R_s_, that represents the solution resistance that is connected in series with the resistor, R_p_, (representing the charge transfer resistance across the passive surface/electrolyte interface), which is parallel to a constant phase element, CPE (Q, relating the properties of the passive surface).

A CPE was introduced in circuit “A” instead of a pure capacitor to consider the nonideal behavior of the passive surface. The polarization resistance of the passive surface, R_p_, was found to be 53.5 KΩ-cm^2^ and the capacitance, C_p_, to be 112.5 μF/cm^2^ for the spray material (CSD) and, for the counterpart ingot alloy (IM390) it was 72.5 KΩ-cm^2^ and 200 μF/cm^2^, respectively. As time increased, significant changes were observed in the impedance diagrams. A second semicircle is clearly distinguished at lower frequencies (inferior to 0.01 Hz) after one day of exposure, which indicates the initiation of pits on the alloy [83]. However, the complex plane also shows that this semicircle remains present and has become more evident as the exposure time is increased. A decrease in the impedance value concerning the time is also observed, which corresponds to the intensification of the pitting corrosion damage during exposure to the chloride media. It is important to note that the equivalent circuit to that shown on Figure 12, circuit A, is used to analyze EIS data for the first hours of immersion; likewise, for the EIS results from 1 to 7 days of exposure, this behavior could be simulated using circuit B, which is proposed for modeling the pitting corrosion mechanism.

The ECC used to describe this EIS response is that shown in circuit “B” (Figure 12). From circuit “A”, the CPE is replaced by a capacitor, C, and an additional RC element was observed in parallel with the passive surface resistance to take into account the impedance of the corroding interface. Therefore, this circuit looks like the pitting model proposed by Mansfeld [89]. Deriving from Mansfeld’s model, the electrical parameters can be estimated, where R_s_ is the solution resistance, R_p_ is the polarization resistance of the passive surface, and C_p_ is its capacitance. At the same time, R_pit_ and C_pit_ are the corresponding parameters for the electrochemical processes occurring in growing pits. All these parameters are functions of *F*, which is the fraction of the pitted area; furthermore, (0 ≤ *F* < 1). W= (K/*F*) (jω)^n^ describes a transmission line element that occurs due to the pitting corrosion mechanism process. Microscopic observations were carried out at the end of the exposure to determine the experimental value of *F* by counting the number of pits and the surface area of each pit.

The values of the electrical parameter of circuit “B” are given in Table 4 and Table 5. The final value of *F* was estimated to be 23% for the cold-spray-deposited (CSD) material with about 85 pits in 0.8 cm^2^ (pitting area, A_pit_ = 0.37 cm^2^) and 37% with more than 200 pits/0.8 cm^2^ (A_pit_ = 0.65 cm^2^) for the ingot alloy (IM390). When pits are not yet initiated, *F* = 0, C_p_ = C_t_ can be assumed according to the following expression C_t_ = C_p_ + *F*C_pit_; but at the first signs of pitting, *F* ≠ 0, and C_t_ depends on *F* as well as the time. C_pit_ can be estimated from the known *F* value at the end of the exposure time, which remains constant during the entire test.

Moreover, the specific experimental pitting capacitance C^o^_pit_ exp can be obtained by normalizing C_pit_ to the total exposure area A = 0.8 cm^2^. According to this expression, C^o^_pit_,-exp = Ct-Cp = FC_pit_; C^o^_pit_ for IM390 (569.33 μF/cm^2^) was slightly smaller than the spray deposit CSD (740 μF/cm^2^). Based on the time dependence of *F*, R^o^_pit_ can also be calculated as a function of time as follows: R^o^_pit_ = R_pit_, exp × A_pit_, but pit growth can be determined from the pit current density *i*_pit_. R^o^_pit_ and *i*_pit_ are related by [R^o^_pit_ = B/*i*_pit_] [50,51], where B is a constant parameter. Thus, changes in R^o^_pit_ with time can be used to determine the pit growth law.

**Figure 13:**
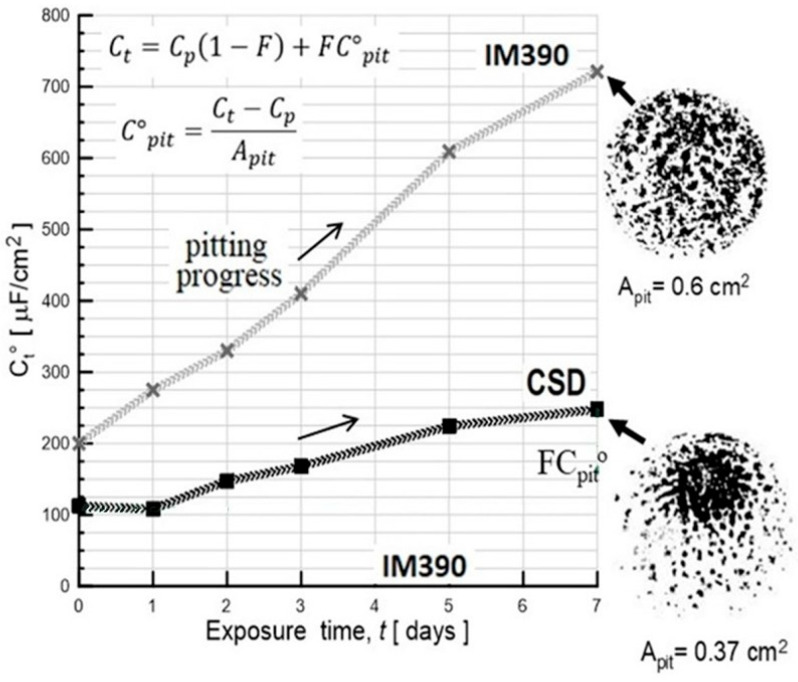
Total capacitance Ct of as-received Al–Si alloys (as-sprayed deposit and IM390-ingot molding) exposed for 7 days in 3.5% NaCl.

The pitting corrosion rate was evaluated by plotting log (1/R^o^_pit_) vs. log t. Figure 13 shows the time dependence of C_t_ and 1/R^o^_pit_ (pit growth rate) for two aluminum–silicon alloys exposed to 3.5 wt.% NaCl during seven days of exposure. A more significant increase in C_t_ was observed for the ingot alloy (IM390), which increased from 200 to 721 μF/cm^2^ during the entire test, resulting in an increase in the pitting tendency for this aluminum sample, while for the cold-spray-deposited (CSD), Ct varied from 112.5 to 248 μF/cm^2^. The results indicate that a larger increase in total capacitance of immersion time of the material tends to corrode more easily by pitting mechanism.

**Figure 14:**
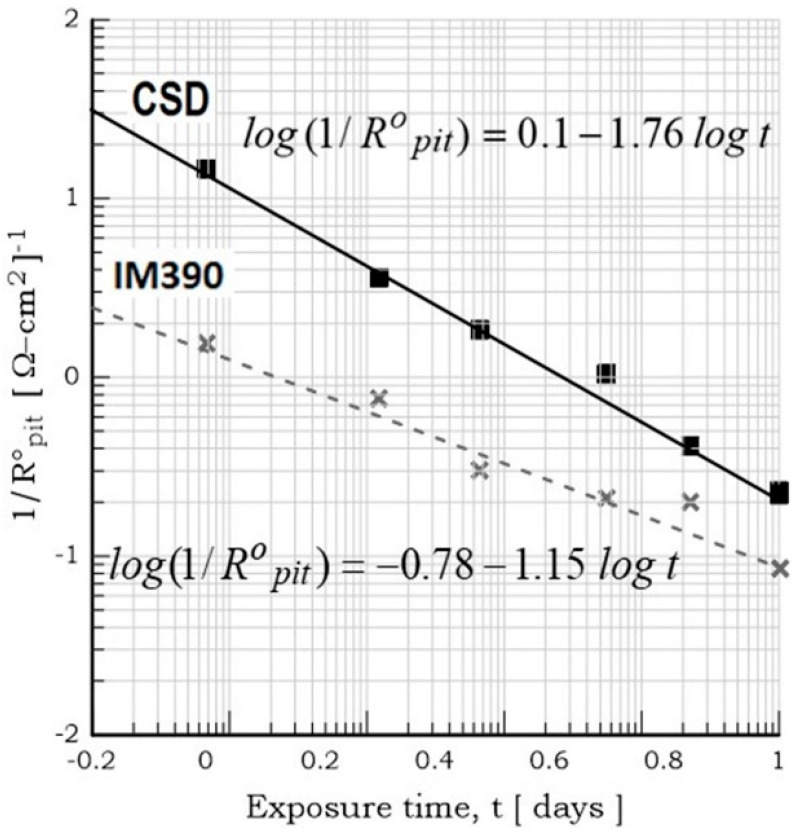
Plot of log (1/R^o^_pit_) vs. log *t* of as-received Al–Si alloys (as-sprayed deposit and IM390-ingot molding) exposed for 7 days in 3.5% NaCl.

Moreover, Figure 14 illustrates a linear relationship between log (1/R^o^_pit_) and log t for both Al–Si alloys. This curve fits to the straight equation y=a+bx, and applying logarithms, an expression that can predict the pitting growth rate is obtained (log (1/R^o^_pit_)= log a + b log t). According to the pitting growth expression, the ingot IM390 alloy results in more pitting damage at the pit propagation rate given by the slope of the straight equation log(1Ropit)=−0.78−1.15 log t than the spray-deposited material (log(1Ropit)=0.1−1.76 log t). Microscopic observation at the end of exposure confirmed the presence of pits and cavities in both materials (Al–Si alloy). Therefore, these results indicate that two types of corrosion processes take place at the same time. One is related to the anodic dissolution $\left(\mathrm{Al}\to {\mathrm{Al}}^{3+}+3e^{-}\right)$ of the Al matrix near the Si particles (hard local cathodic sites), resulting in small pits, and the other proceeds around the Si particle until the detachment of the particle, leaving a big cavity due to the intensification of corrosion attack, as represented in Figure 9.

**Figure 15:**
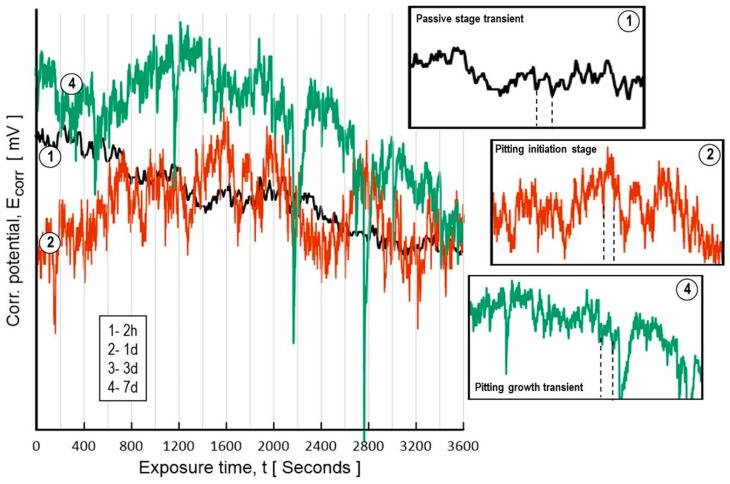
Potential transients of as-received spray deposit CSD exposed for 7 days in 3.5% NaCl. Note: The insets show the amplitude of the transient magnitude for pitting corrosion. Transient 1 for 2 h of immersion, 2 for 1 day, 3 is not shown for clearness of the plot and 4 for 7 days.

In general, high silicon alloying in aluminum causes pitting corrosion in chloride solutions, which is monitored using the EIS technique by the occurrence of a second semicircle at lower frequencies with a more significant increase in Ct during the entire test time, and a severe decrease in |Z| was also related to the pit initiation. In addition to transient fluctuations in voltage as a function of time, as denoted in Figure 15, the spectra show different shapes of the transients, where a smooth signal waveform indicates a passive state by an oxide layer (Transient 1 and 2 h of exposure time in NaCl), but a more significant increase in potential intensity and a very noisy signal can be characterized by pitting corrosion occurrence in the Al matrix due to silicon dispersion. Transient 2 shows the behavior during the pitting initiation process at one day of exposure to NaCl, and Transient 4 responds to the signal for seven days of NaCl exposure, which implies pitting growth kinetics.

### 3.3. Surface Treatment as Corrosion Protection

#### 3.3.1. Potentiodynamic Polarization Test of the Treated Condition

Anodizing treatment by sulfuric acid is an oxidation process that protects the metal surface against pitting corrosion; consequently, a thick oxide layer can be obtained on the aluminum surface, which can control the Al-α dissolution in many corrosive environments. According to other research works [82,90], the anodic oxide layer is entirely formed of a hexagonal columnar structure with open pores, but in the presence of Si particles, this film is always distorted by superficial defects like cracks, thinning, or even its absence in localized regions. However, this layer substantially needs a hydrothermal sealing treatment to fill the internal structure with aluminum hydroxide compounds.

**Figure 16:**
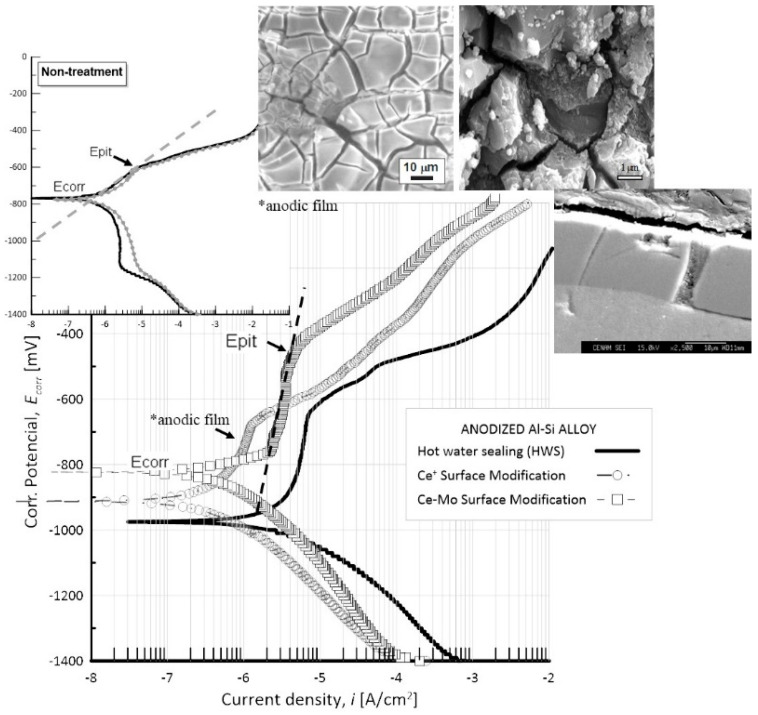
Anodic polarization curves for the H_2_SO_4_ anodized cold-spray-deposited Al–Si alloy that was hydrothermally sealed in different reagents: HWS; CeSM; and Ce–MoSM. Inset a SEM micrographs show anodic film morphology after hydrothermal sealing.

In this experimental section, electrochemical results in terms of anodic polarization are shown for Al–Si spray-deposited (CSD substrate) anodized in an electrolytic cell containing a concentrated solution of H_2_SO_4_. Immediately, a hot sealing procedure through only boiling water was applied, and other anodized samples were sealed in hot baths containing cerium or molybdenum ions. This treatment is named here as surface modification by chemical conversion. Figure 16 displays the experimental results of the anodic polarization curves during a particular exposure period at the corrosive solution of 35 g/L NaCl for anodized Al–Si spray-deposited samples (CSD). Sealed in three different conditions, hydrothermal sealing in hot water HWS or in boiling solution containing ions of Ce^3+^ CeSM and with Mo^6+^ Ce–MoSM, non-anodized samples are also shown as a comparison.

When the anodized specimen is thermally sealed with Ce^3+^ solution, an increase in surface activity (E_corr_) from −967 to −823 mV and an augment in pitting potential (E_pit_) from −530 mV to −491 mV were found, due to cerium hydroxide precipitating into the defects, microcracks, and pore mouths of the anodic oxide film. In addition, silicon particles dispersed on the Al-α matrix can produce deep cracks, discontinuities, or local breakdown of the anodic film, which are suggested by SEM microscope examination (micrographs inset in Figure 16). Sealing the anodic pore structure with only aluminum hydroxide compounds that were precipitated from a hot water solution deteriorated the corrosion potential by about −975 mV. A slightly improved pitting potential value of about −626 mV with respect to the nontreated sample was observed. Furthermore, the corrosion rate was almost 70 times diminished, but several pits on the surface were seen through SEM examination (see inset in Figure 16). Concerning the use only of Ce^3+^ solution, the improvement in corrosion values was not clearly observed in comparison with Ce–Mo sealing. Generally speaking, changes in the slope rate in the anodic region were observed for samples HWS, CeSM, and Ce–MoSM, which indicate passive behavior.

The anodic curve indicated by Ce–Mo has a lower current density than the other curves, although its E_corr_ is more active, and its E_pit_ is about in the −491 mV range. Nonetheless, an increase in the pitting potentials of anodizing samples for the Al–Si spray-deposited E_pit_ up to 100 mV, approximately concerning this initial stage, was noticed. For the surface conversion treatment with CeCl_3_ and Na_2_MoO_4_, a tendency of corrosion rate (*i*_corr_) decreased from 1.009 to 0.111 mA. For the IM390 casting alloy, an improvement after anodizing process was not noticed due to the coarser silicon crystals on the Al matrix.

#### 3.3.2. Impedance Test of the Treated Condition

**Figure 17:**
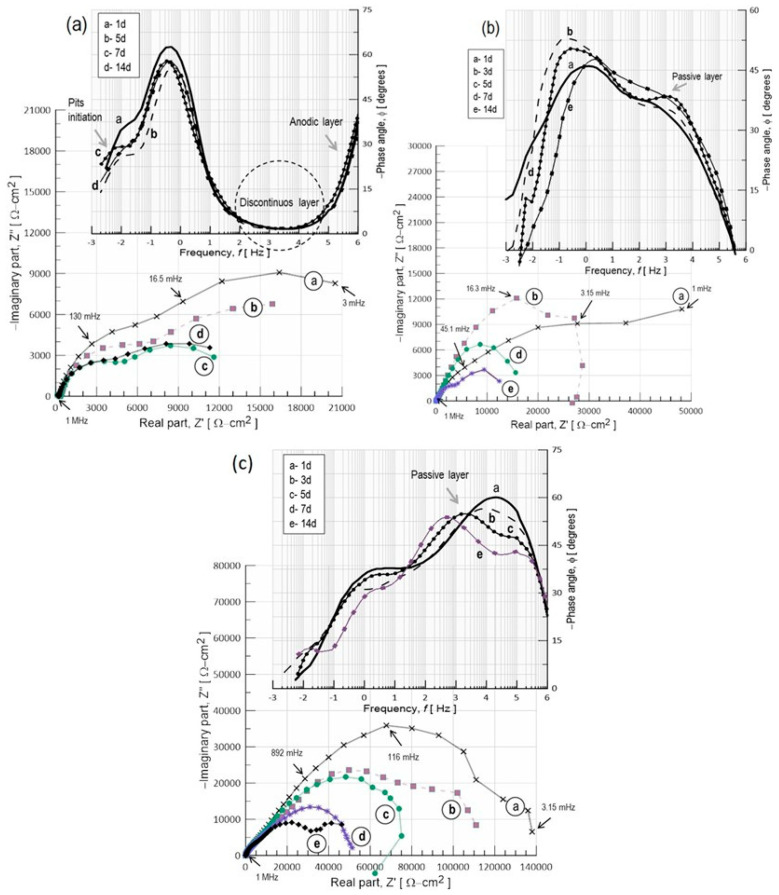
Impedance results for H_2_SO_4_ anodized spray-deposited Al–Si alloy and passivated in (**a**) hot water for 60 min, (**b**) 10 mM CeCl_3_ for 20 min, and (**c**) 10 mM CeCl_3_ and 10 mM Na_2_MoO_4_ for 20 min, tested in 3.5% NaCl as a function of exposure time.

The impedance spectra in the Nyquist form and Bode plots (phase angle vs. frequency) of the anodized spray-deposited (CSD) Al–Si samples treated with different sealing reagents are displayed in Figure 17 as a function of immersion time in 35 g/L NaCl. The anodized surface was treated as sealing in hot water, HWS (Figure 17a), for 60 min or in a boiling solution containing 10 mM CeCl_3_ for 20 min (cerium surface modification, CeSM, Figure 17b), or in a hot solution of 10 mM CeCl_3_ for 20 min and immersed in 0.1M Na_2_MoO_4_ (molybdenum surface modification), Ce–Mo (Figure 17c), to improve pitting corrosion resistance. The impedance spectra for the CSD sealed in hot water (HWS) displayed two well-defined semicircles in the entire frequency, measured after two hours of immersion (Figure 17a).

**Figure 18:**
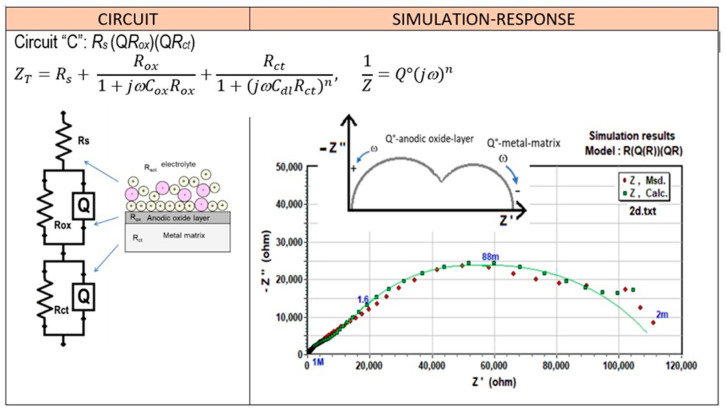
Representation of the simulation response of the equivalent electrical circuits (EECs) used to model the impedance behavior of H_2_SO_4_ anodized cold-spray-deposited Al–Si alloy exposed for 7 days in 3.5% NaCl.

The first loop at high frequencies indicates the presence of a physical barrier layer, in this case, the sealed porous aluminum oxide layer, but it is known that Si particles break down the continuity of this layer, which causes local cracks and detachment of the oxide layer. Thus, the second loop at lower frequencies is probably associated with the infiltration of the electrolyte [Cl_s_^−^] through the film pores and defects, and a decrease in the impedance value was also observed with increasing immersion time. This suggests that the commonly used hot water sealing (HWS) process is not recommended for this kind of aluminum-anodizing alloy, where pits were observed. Nevertheless, a decrease in the phase angle of about −1.66 degrees at 3.23 Hz indicates water uptake through open pores and defects on the anodic oxide layer. By using the ECC circuit model marked with the letter “C”, as shown in the inset of Figure 18, the impedance behavior is very reasonably described.

The simulation results show that the increase in coating capacitance from 4 to 73.6 mF/cm^2^ and its resistance (*R_po_*) was estimated to be about 200 Ω-cm^2^ after 14 days of immersion. This indicated that the aluminum anodized layer was not entirely sealed by resistive aluminum oxide/hydroxide compounds, which promotes the easy dissolution of the protective oxide layer by Cl^−^ attack, causing pits on the Al-α matrix. The capacitance of the electrochemical double layer (*C_dl_*) increases sharply from 313.4 to 981.89 mF/cm^2^, and the polarization resistance (*R_p_*) was significantly lower than the untreated alloy (~6.4 KΩ-cm^2^), suggesting that the pitting growth process began after 1 or 2 days of exposure. After one week of immersion, the pitted area fraction was about 0.23 cm^2^. The cause for this significant increase in *C_dl_* might be that the anodic oxide film on the surface was activated during immersion in hot water. This makes the surface rough and highly susceptible to pitting corrosion when it is exposed to NaCl solution.

**Figure 19:**
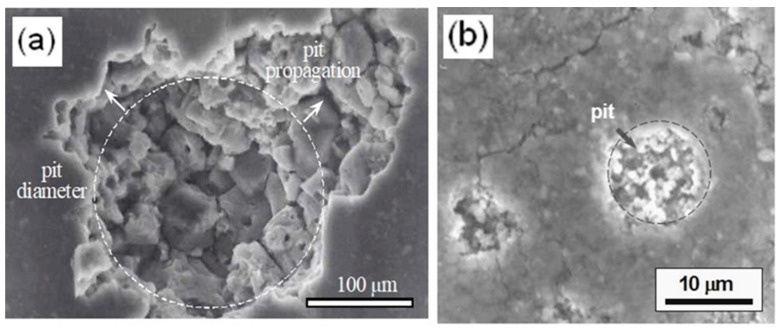
SEM images showing the surface appearance of Al–Si alloy after exposure to 3.5% NaCl for 7 days. (**a**) pit propagation on Al matrix, (**b**) spherical pit distribution on the Al matrix.

**Figure 20:**
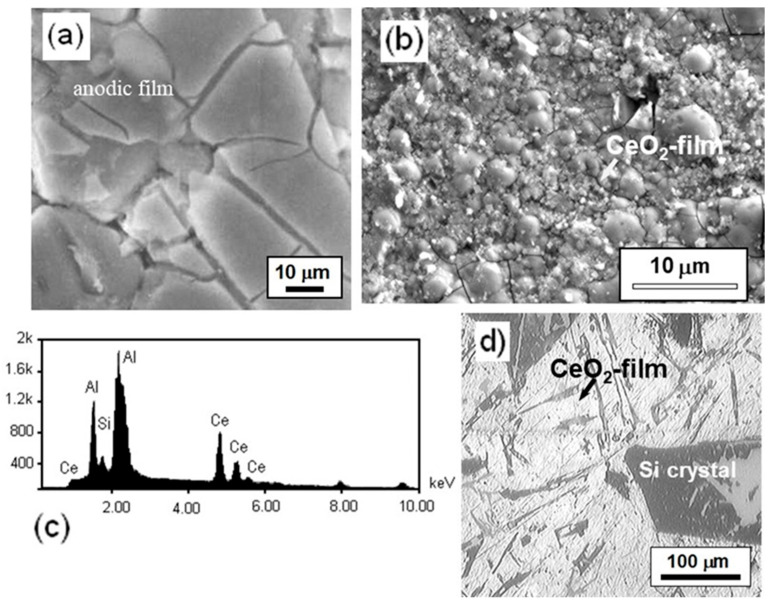
SEM images of Al–Si alloy showing the surface appearance of cerium surface modification process (CeSM) after exposure to 3.5% NaCl for 7 days. (**a**) hot water sealing HWS, (**b**) cerium sealing CeSM, (**c**) EDS shows Ce ions on the anodic film, (**d**) CeSM treatment applied on the IM390 alloy.

Different results were observed when the anodic surface was modified with cerium ions. Very stable two-time constants at the first days of immersion were observed, but at time t ≥ 5 days, there was an increase in their capacitance from 37 to 3 mF with impedance losses of 10^5^ to 10^3^ Ω-cm^2^, indicating adsorption of NaCl electrolyte on the anodic film. Finally, a few pits were detected with salt residues that were rich in Ce, which possibly acts as an anode and creates an alkaline environment that accelerates the dissolution of the anodic coating, as indicated in Figure 19. Better results were found using the cerium and molybdenum surface modification (Ce–Mo). The impedance behavior of this treatment is like that shown in Figure 17c. Two capacitive semicircles were still observed in the whole immersion period. The shape of these semicircles indicated that cerium produced a very effective sealing of the pores or cracks on the anodized spray-deposited Al–Si alloy (CSD). Thus, no pits were observed due to the precipitation of compounds of CeO_2_/Ce(OH)_2_, which decreases the rate of oxygen reduction (O_2_ + 2H_2_O + 4e^−^ →4OH^−^) at cathode sites that cause an acceleration of the oxidation process from Ce^3+^ to Ce^4+^ to form a cerium hydroxide compound (4Ce^3+^ +O_2_^+^ 2H_2_O_(l)_ + 4OH^−^→4Ce(OH)_2_^2+^) with molybdenum precipitates, shown in Figure 20.

## 4. Conclusions

The microstructures of the as-sprayed products involved in this research presented in Figure 5 and Figure 6 proved that regardless of the processing route spray atomization and co-deposition, products exhibited much finer structure than conventionally cast alloy IM390. Generally, two notable microstructural features in as-sprayed deposits and extrudates were the particulate-like Si phase and equiaxed grains. In correlating the observed equiaxed microstructure with the dynamic procedures in spray deposition, we can conclude that a semiliquid/semisolid layer developed on the deposition surface. The impact of solid and semisolid droplets on the deposition surface provided dendrite arm fragments, which may act as nucleation centers for equiaxed grains. Pits initiated surface oxide flaws, often corresponding to metallic surface heterogeneities.

In the Al_2_O_3_-containing composites, galvanic coupling was detected to a limited extent between the second phases precipitated (or silicon) and the matrix. According to the report, the big silicon particles were slightly oxidized during the anodizing process and acted as oxygen reduction cathodic sites, whereas smaller silicon particles up to about 5 µm became occluded.

The big silicon crystals (~150 µm) segregated as blocks from the sample IM390 as cathodes; therefore, these particles increased the matrix corrosion rate and its corrosion potential up to *i*_corr_ = 24.7 µA/cm^2^, E_corr_ = −1320 mV, and E_pit_ = −670 mV, respectively, making the material more active. Even though there was passivity, a high corrosion rate in the passive stage was observed. The corrosion pitting potential, E_pit_, decreased from −667 mV to −410 mV when the alloy was sealed with cerium Ce^+3^ solution. This diminution is likely due to the flaws from the passive layer present in silicon crystal borders.

In turn, the extrudate ExCSD had smaller silicon particles (~12 µm) with a lower corrosion rate, *i*_corr_ = 1.2 µA/cm^2^, a corrosion potential of E_corr_ = −966 mV, and a pitting potential of E_pit_= −587 mV. The cathodic oxygen reduction was considered to be the main driving force for the corrosion process, and the cathodic sites were silicon particles.

In NaCl solutions, the alloys’ E_pit_ values were not significantly affected by the aerated solution (100 mV). *i*_corr_ and the localized corrosion rate increased with the size of the volume fraction of silicon particles.

Cerium–molybdenum sealing provided better corrosion protection than hot water sealing. This result was attributable to the passivating effects of Ce(III) deposited in the pores of the outer oxide layer. The anodizing and CeCl_3_ and Na_2_MoO_4_ treatment of aluminum as-sprayed deposits and extrudates gave some protection; nonetheless, cerium solutions represent less toxic media than chromates.

## Figures and Tables

**Table 1:** Processing characteristics of spray deposits and their extrudates.

Deposit	Gas/Metal Ratio [m^3^/Kg]	Extrudates	Diameter	Extrusion Ratio
HSD	2.5	ExHSD	2.54	4:1
CSD	4	ExCSD	2.54	4:1

**Table 2:** Chemical composition of Al–Si preforms and their extrudates, in wt%.

Preforms	Si	Cu	Mg	Fe	Al
HSD	16.70	4.55	0.56	0.4	Balance
CSD	16.54	4.4	0.56	0.35	Balance
Extrudates					
ExHSD	16.10	4.25	0.50	0.30	Balance
ExCSD	16.30	3.12	0.40	0.43	Balance

**Table 3:** Electrochemical parameters of Al–Si samples in 3.5% NaCl, without surface treatment.

Parameters	E_corr_ [mV]	E_pit_ [mV]	*i*_corr_ [μA/cm^2^]
Extrudate-ExCSD	−966	−587	1.2
Preform-CSD	−1030	−598	2.47
Preform-HSD	−1210	−579	14.05
IM390	−1320	−670	24.7

**Table 4:** Impedance parameters of spray-deposited Al–Si (CSD sample) during exposure for 7 days in 3.5% NaCl, without surface treatment.

Time [Days]	C_t_ ^o^ [µF/cm^2^]	R_p_/(1-F) [kΩ-cm^2^]	R_pit_/(F) [kΩ-cm^2^]	K/F [kΩ-cm^2^]	α
0	112.5	53.5	-	-	0.68
1	108.3	52.9	13.2	1.53	0.71
2	147.5	33.8	10.5	1.71	0.95
3	168	26.5	8.91	2.39	0.96
5	224.3	23.1	6.24	2.16	0.95
7	248	12.9	2.83	1.79	0.96

**Table 5:** Results of pitting analysis of unprotected aluminum–silicon alloys after exposure to 3.5% NaCl for 7 days using an optical microscope at 30× according to ASTM-G46 [49].

Alloy	C_pit_^o^ [µF/cm^2^]	*F* [68]	A_pit_ [cm^2^]	Density of Pits [68]	No. of Pits	Pitting Time
CSD	740	22.8	0.37	22.8	<80	1–2 days
IM390	569.33	36.98	0.65	50.5	>200	2 h

## Data Availability

The data that support the findings of this study cannot be made freely available. Requests for access to these data should be made to the corresponding author.
